# The Association of Depression, Loneliness, and Internet Addiction Levels in Male Bachelor of Medicine, Bachelor of Surgery (MBBS) Students With Androgenetic Alopecia Male Pattern Baldness in a Medical College in Kolar, India

**DOI:** 10.7759/cureus.35607

**Published:** 2023-02-28

**Authors:** Harish Prasanna, Rajashekar T S, Suresh Kumar K, Athish KK, Madhu Kiran, Meghana Reddy

**Affiliations:** 1 Dermatology, Venereology, and Leprosy, Sri Devaraj Urs Academy of Higher Education and Research, Kolar, IND

**Keywords:** androgenetic alopecia, male pattern baldness, depression, loneliness, internet addiction, male medical students

## Abstract

Introduction

The genetically determined progressive process that causes a gradual conversion of terminal hair into vellus hair is known as androgenetic alopecia (AGA). AGA male pattern baldness is very common among male medical students whose self-image is severely deteriorated by AGA and this affects the quality of their professional career. Hence, the assessment of the association of depression, loneliness, and internet addiction levels of male Bachelor of Medicine, Bachelor of Surgery (MBBS) students with AGA male pattern baldness is essential to improving academic and professional performance.

Aims and objectives

The aim of this study is to evaluate the effects of AGA male pattern baldness and its severity on depression, loneliness, and internet addiction levels of male medical students in Kolar.

Materials and methods

This questionnaire-based cross-sectional study was conducted among 100 male MBBS students from Sri Devaraj Urs Medical College in Kolar with AGA male pattern baldness of varying grades. The participants were selected through simple random sampling from July 2022 to November 2022 with their prior informed consent. Students' AGA severity was evaluated clinically using the Norwood-Hamilton Classification. Their levels of depression, loneliness, and internet addiction were assessed using the standardized Beck Depression Inventory (BDI) scale, University of Carolina Los Angeles - Loneliness Scale (UCLA-LS), and Young Internet Addiction Test - Short Form (YIAT-SF), respectively, and one-way analysis of variance (ANOVA) was used to ascertain the statistical significance between the means of BDI, UCLA-LS, and YIAT-SF with the severity of AGA. Chi-square/Fisher Exact test was used to find the significance of study parameters on a categorical scale between two or more groups. Significance was assessed at a 5% level of significance.

Results

The mean of BDI (17.38, 25.11, 34.62, 41.25, 51.00), UCLA-LS (18.72,27.51,36.69,43.5,49.00), and YIAT-SF (20.51, 31.77, 50.31, 60.25, 72.00) scores in each of the AGA grades from Grade I to Grade V in our study showed that these scores increase along with an increase in the severity of AGA and are statistically significant. The frequency distribution of male medical students with varied degrees of AGA and the level of depression, loneliness, and internet addiction levels assessed by the BDI, UCLA-LS, and YIAT-SF showed a robust and statistically significant association between the severity of AGA and the severity of depression, loneliness, and internet addiction levels.

Conclusion

The current study showed that there is a statistically significant association of depression, loneliness, and internet addiction levels in male MBBS students with AGA male pattern baldness.

## Introduction

One of the distinguishing features of mammals is hair. It has always had a significant psychological and sociological role in shaping a person's appearance and personality. Different hairstyles and shaving techniques reflect the cultural origins of people. Additionally, it conveys the individual's standing and unique personality. Due to media and public hype surrounding hair, having too little hair, as in alopecia, is socially and cosmetically unacceptable [1]. In both social and sexual communication, hair is crucial [2].

Androgenetic alopecia (AGA), the most prevalent kind of hair loss affecting approximately 70% of adult males and 50% of adult females, is a genetically set progressive process that gradually transforms terminal hair into vellus hair [1,2]. Involving both hormonal and hereditary variables, AGA is a benign illness that can have a profound psychological impact on a person [1]. Due to an overactive reaction to androgens, the pattern of AGA is a genetically defined condition. It is characterized by the gradual loss of the scalp's terminal hair at any point after puberty, with a distinctive pattern in males. The vertex and frontotemporal regions of males have the most pronounced hair loss [3].

Past research indicates that AGA may also have psychosocial side effects, such as depression, low self-esteem, altered self-image, and fewer frequent pleasurable social interactions. People with AGA frequently believe that their alopecia is a serious condition that will undoubtedly have negative effects on their lives [1].

College students who are adolescents and young adults are in a crucial stage of psychological development, and their psychological state is unstable. College students with AGA might have to deal with the extra stress of hair loss, which could put a significant strain on their capacity for coping [2]. The significance of depression in AGA has been demonstrated by a number of studies. College students who are depressed often withdraw from others and start engaging in certain addictions. The use of the internet has increased rampantly not only in India but also globally. The internet has taken the top spot as a necessity in our daily lives. It is widely used all throughout the world, particularly among adolescents and young people. As a result, they cut themselves off from their surroundings and live by themselves in their own lonely world using the internet. It has developed into a problematic use that is connected to a number of emotional and physical side effects. Because of this, the lives of today's youth are a living nightmare of depression, loneliness, and internet addiction [4].

Numerous studies have demonstrated that during their time in medical school, students endure heightened levels of stress. They are under a lot of stress because of the sacrifices they must make on a personal and social level to maintain good academic outcomes in a setting of intense competition. Researchers discovered that medical students' stress levels were ostensibly higher than those of non-medical professional students. Stress can cause problems with one's physical and mental health. Drugs, painkillers, alcohol, the internet, smoking, and eating may be used to cope, but these are unproductive and may make the stress worse. It may also cause depression and anxiety [5]. Internet addiction has becoming increasingly common among depressed people in today's digital age [6]. This study has been carried out because there is a dearth of literature on the relationship between depression, loneliness, and internet addiction levels in male medical students with AGA male pattern baldness who are in a particularly sensitive stage for body image and social relationships.

## Materials and methods

Methodology

This questionnaire-based cross-sectional study was conducted among 100 male Bachelor of Medicine, Bachelor of Surgery (MBBS) students from Sri Devaraj Urs Medical College in Kolar, Karnataka, India, with AGA male pattern baldness of varying grades, who were selected through simple random sampling from July 2022 to November 2022 with their prior informed consent. Students' AGA severity was evaluated clinically using the Norwood-Hamilton classification. The questionnaires that all participants in this study completed included questions about their socioeconomic status as well as assessments of their levels of depression, loneliness, and internet addiction using the standardized Beck Depression Inventory (BDI) scale, University of Carolina Los Angeles - Loneliness Scale (UCLA-LS), and Young Internet Addiction Test - Short Form (YIAT-SF), respectively.

Statistical analysis

Data from the study was recorded into an Excel datasheet (Microsoft Corporation, Redmond, Washington, United States). The data analysis was done using IBM SPSS Statistics for Windows, Version 22.0 (2013; IBM Corp., Armonk, New York, United States) and R 3.2.2 (2015; R Foundation for Statistical Computing, Vienna, Austria). Results on continuous measurements were presented as mean ± SD (min-max) and results on categorical measurements were presented as number (%). To ascertain whether there were any statistically significant differences between the means of three or more independent (unrelated) groups, the one-way analysis of variance (ANOVA) was used. Chi-square/Fisher Exact test was used to find the significance of study parameters on a categorical scale between two or more groups. Significance was assessed at a 5% level of significance.

## Results

One hundred MBBS students with varied degrees of male pattern AGA participated in the current study. Table 1 shows the frequency of distribution of students based on the MBBS year of the current study.

**Table 1:** MBBS year-wise frequency distribution of students MBBS: Bachelor of Medicine, Bachelor of Surgery

MBBS Year	No. of Students	%
I	22	22
II	20	20
III	28	28
IV	30	30
Total	100	100

The students in our study ranged in age from 18 to 24 years and had a mean age of 20.41. The MBBS students in our study had AGA male pattern baldness grades ranging from Grade I to Grade V using the Norwood-Hamilton scale. Table 2 shows the frequency of distribution of students based on AGA grading with the MBBS year.

**Table 2:** Androgenetic alopecia grading frequency distribution of students MBBS: Bachelor of Medicine, Bachelor of Surgery

Androgenetic Alopecia Grading	MBBS Years				Total
	I	II	III	IV	
1	18 (81.8%)	16 (80%)	7 (25%)	6 (20%)	47 (47%)
2	4 (18.2%)	4 (20%)	16 (57.1%)	11 (36.7%)	35 (35%)
3	0 (0%)	0 (0%)	5 (17.9%)	8 (26.7%)	13 (13%)
4	0 (0%)	0 (0%)	0 (0%)	4 (13.3%)	4 (4%)
5	0 (0%)	0 (0%)	0 (0%)	1 (3.3%)	1 (1%)
Total	22 (100%)	20 (100%)	28 (100%)	30 (100%)	100 (100%)

The current study's data indicate that the incidence of AGA severity increases with the year of MBBS; 94% of the students in our study had stress in their day-to-day activities. The mean of BDI, UCLA-LS, and YIAT-SF scores in each of the AGA male pattern baldness grades from Grade I to Grade V in our study shows that these scores increase along with an increase in the severity of AGA and are statistically significant. The severity of the AGA graded from Grade I to Grade V in our study, was compared to the mean of the BDI, UCLA-LS, and YIAT-SF scores in Table 3.

**Table 3:** Comparison of BDI, UCLA-LS, and YIAT-SF with the severity of AGA male pattern baldness BDI: Beck's Depression Inventory; UCLA-LS: University of Carolina Los Angeles - Loneliness Scale; YIAT-SF: Young Internet Addiction Test - Short Form; AGA: androgenetic alopecia

Variables	Androgenetic Alopecia Grading					Total	P-value
	1	2	3	4	5		
Beck's Depression Inventory	17.38±5.56	25.11±7.81	34.62±5.3	41.25±5.12	51.00±0.00	23.62±9.73	<0.001**
University of Carolina Los Angeles - Loneliness Scale	18.72±7.4	27.51±8.09	36.69±9.33	43.5±6.76	49.00±0.00	25.43±10.87	<0.001**
Young Internet Addiction Test - Short Form	20.51±7.77	31.77±10.01	50.31±11.09	60.25±3.59	72.00±0.00	30.43±15.18	<0.001**

The frequency distribution of students with varied degrees of AGA male pattern baldness and the level of depression assessed by BDI showed a robust and statistically significant association between the severity of AGA and the severity of depression. Table 4 lists the results of the frequency distribution of pupils with varying levels of AGA and the degree of depression determined by BDI.

**Table 4:** Distribution of students' levels of depression as measured by the BDI and AGA in varying degrees BDI: Beck's Depression Inventory; AGA: androgenetic alopecia

Beck's Depression Inventory Score	Androgenetic Alopecia Grading					Total	P-value
	1	2	3	4	5		
Normal (1-10)	0 (0%)	1 (2.9%)	0 (0%)	0 (0%)	0 (0%)	1 (1%)	<0.001**
Mild depression (11-16)	26 (55.3%)	4 (11.4%)	0 (0%)	0 (0%)	0 (0%)	30 (30%)	
Borderline depression (17-20)	9 (19.1%)	3 (8.6%)	0 (0%)	0 (0%)	0 (0%)	12 (12%)	
Moderate depression (21-30)	11 (23.4%)	20 (57.1%)	2 (15.4%)	0 (0%)	0 (0%)	33 (33%)	
Severe depression (31-40)	1 (2.1%)	6 (17.1%)	9 (69.2%)	2 (50%)	0 (0%)	18 (18%)	
Extreme depression (40-63)	0 (0%)	1 (2.9%)	2 (15.4%)	2 (50%)	1 (100%)	6 (6%)	

There was a strong and statistically significant association between the severity of AGA and the severity of loneliness, as evidenced by the frequency distribution of students with various degrees of AGA male pattern baldness and the level of loneliness measured by the UCLA-LS. The frequency distribution of students with varying levels of AGA and the UCLA-LS scores of their level of loneliness are shown in Table 5.

**Table 5:** Distribution of students' levels of Loneliness as measured by the UCLA-LS and AGA in varying degrees UCLA-LS: University of Carolina Los Angeles - Loneliness Scale; AGA: androgenetic alopecia

University of Carolina Los Angeles - Loneliness Scale Score	Androgenetic Alopecia Grading					Total	P-value
	1	2	3	4	5		
Normal (1-14)	12 (25.5%)	5 (14.3%)	2 (15.4%)	0 (0%)	0 (0%)	19 (19%)	<0.001**
Mild degree of loneliness (15-29)	25 (53.2%)	13 (37.1%)	1 (7.7%)	0 (0%)	0 (0%)	39 (39%)	
Moderate degree of loneliness (30-44)	8 (17%)	13 (37.1%)	7 (53.8%)	1 (25%)	0 (0%)	29 (29%)	
Severe degree of loneliness (45-60)	2 (4.3%)	4 (11.4%)	3 (23.1%)	3 (75%)	1 (100%)	13 (13%)	

The frequency distribution of students who displayed various levels of the AGA male pattern baldness and the degree of internet addiction determined by the YIAT-SF revealed a strong and statistically significant association between the severity of AGA and the severity of internet addiction. The frequency distribution of students with varying degrees of AGA and the degree of internet addiction as indicated by the YIAT-SF are shown in Table 6.

**Table 6:** Distribution of students' levels of internet addiction as measured by the YIAT-SF and AGA in varying degrees YIAT-SF: Young Internet Addiction Test - Short Form; AGA: androgenetic alopecia

Young Internet Addiction Test - Short Form Score	Androgenetic Alopecia Grading					Total	P Value
	1	2	3	4	5		
Normal (0-30)	41 (87.2%)	14 (40%)	0 (0%)	0 (0%)	0 (0%)	55 (55%)	<0.001**
Mild (31-49)	6 (12.8%)	19 (54.3%)	6 (46.2%)	0 (0%)	0 (0%)	31 (31%)	
Moderate (50-79)	0 (0%)	2 (5.7%)	7 (53.8%)	4 (100%)	1 (100%)	14 (14%)	
Severe (80-100)	0 (0%)	0 (0%)	0 (0%)	0 (0%)	0 (0%)	0 (0%)	

## Discussion

As the guardians of the future generation, medical students' health must be given the utmost priority. Due to their heavy academic load, personal issues, and the need to learn to save patients' lives, students in medical school experience a great deal of stress. Additionally, there is a very high prevalence of AGA male pattern baldness among medical students. In terms of interpersonal sensitivity, the psychological circumstances of medical students with AGA male pattern baldness are much worse than those of students in other professions [2,5]. Medical students' self-image is greatly impacted by AGA, which also disrupts their social lives. When college students are depressed, they frequently isolate themselves, start engaging in specific addictions, and withdraw from others [4]. Therefore, this study was conducted to fill the gap in research on the relationship of depression, loneliness, and internet addiction levels with AGA male pattern baldness in male medical students who are at a particularly vulnerable age for body image and social relationships.

In the current study, 94% of the students with AGA male pattern baldness reported experiencing stress from their daily activities. This result is in line with the elevated stress levels observed in the medical students as a whole in the study carried out by Jafri et al. in which they recorded 75.6% of medical students as having stress [5]. The influence of AGA is a factor that adds to the frequency of stress in male medical students.

The severity of AGA grading increased along with the degrees of depression in our study population. Mirza et al. recorded the rising prevalence of depression among medical students in general [7]. Wang et al. reported the prevalence of depression among college students with AGA male pattern baldness, although they did not focus on medical students specifically [2]. In the male medical students we studied, we found that AGA male pattern baldness was associated with depression.

College students who are sad have a propensity for isolation and a life devoted to various addictions [4,8]. Due to their loneliness, many college students who are living in the digital age get glued to the internet. According to a study by Zhang et al., the prevalence of internet addiction among medical students is about five times higher than the general population [9]. This is consistent with the current study's findings, which show that male medical students with AGA have higher degrees of loneliness and higher levels of internet addiction. The impact of the severity of AGA on the level of loneliness and internet addiction in male medical students has implied the downregulation of self-image due to AGA male pattern baldness, leading them to dwell in loneliness and invest in various addictions, especially internet addiction.

According to Perera et al., AGA male pattern baldness is the most typical cause of hair loss in medical students. They revealed that AGA male pattern baldness in medical students indicates a serious negative impact on their lives in terms of their general self-esteem and frame of mind, which seriously affects their psychic nature and results in depression, loneliness, and internet addiction [10]. This result is in accordance with that of our study, which showed a statistically significant association between the severity of AGA in male medical students and degrees of depression, loneliness, and internet addiction.

## Conclusions

The results of this study show that there is a statistically significant association of depression, loneliness, and internet addiction levels with AGA male pattern baldness in male MBBS students. Medical students are under constant stress during their medical school life. Male medical students have a relatively higher incidence of AGA male pattern baldness than the general population. AGA is aesthetically unappealing and has major psychological morbidity in male medical students. Because they are the doctors of tomorrow, medical students' health is of the utmost significance. Therefore, early detection and timely intervention have to be done to combat depression, loneliness, and internet addiction in male medical students with AGA.
